# Kidney failure of unknown cause: a call to admit our uncertainty

**DOI:** 10.1590/2175-8239-JBN-2022-0134en

**Published:** 2023-04-28

**Authors:** Maristela Böhlke

**Affiliations:** 1 Universidade Católica de Pelotas, Hospital Universitário São Francisco, Programa de Pós-Graduação em Saúde e Comportamento, Pelotas, RS, Brazil.

Since 2010, the Brazilian Society of Nephrology has been collecting and publishing data on chronic kidney failure (CKF) treated by dialysis in Brazil. Hypertensive nephrosclerosis has been reported by Brazilian dialysis units as the most prevalent primary kidney disease leading to CKF^1^.

**Figure 1:**
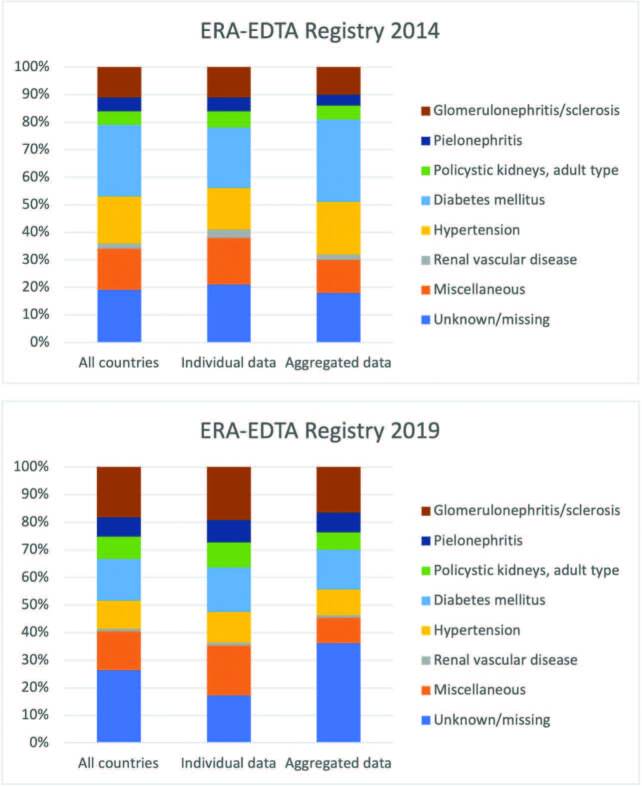
Primary kidney diagnosis of patients in RRT in Europe, 2014 (ERA-EDTA Registry 2014); Primary kidney diagnosis of patients in RRT in Europe, 2019 (ERA-EDTA Registry 2019).

Reports from the United States Renal Data System have also long described hypertension as the second most frequent cause of CKF^2^. The ERA-EDTA Registry 2019 Annual Report, however, attributed only 10% of the cases of kidney replacement therapy (KRT) in Europe to hypertension^3^, a decrease from the 17% reported in 2014^4^, with a proportional increase in unknown primary diagnosis from 19% to 26%^3,4^. (Figure 1)

In 2020, we carried out an in-depth review of the medical records of 210 patients with CKF treated by dialysis or kidney transplantation at a university hospital in southern Brazil. Patients had been followed up from the early stages of chronic kidney disease at the university’s outpatient clinic, undergoing a thorough workup, including kidney biopsy when indicated. Genetic investigation, however, was not available.

Most patients had diabetic kidney disease (29%), 24% of the sample had unknown primary kidney disease (most with shrunken kidneys at presentation), 20% had non-diabetic glomerular disease, and 15% had urological abnormalities as the cause of kidney failure. Only one patient with clinical history of refractory hypertension and histological findings of glomerular thrombotic microangiopathy (no evidence of complement, immune-mediated or ADAMTS13-linked diseases) and another patient with histology suggestive of glomerulosclerosis were classified as having hypertensive kidney disease.

The nephrology scientific community has long recognized that hypertensive nephrosclerosis is a less common cause of CKF than previously thought^5^.

This diagnosis is usually established on clinical grounds alone in patients with hypertension, chronic kidney disease, and low-level or absent proteinuria. However, several other primary diseases can have the same presentation, including potentially treatable inflammatory glomerulopathies or genetic disorders. As an example, glomerulosclerosis associated with variants of the APOL1 gene (now included in the category of podocytopathies, segmental and focal glomerulosclerosis subtype) was considered, before the description of the risk alleles, as a more aggressive presentation of hypertensive nephrosclerosis affecting patients with African ancestry^5^.

In conclusion, reporting unknown primary kidney disease as if it were hypertensive nephrosclerosis masks the reality that we often do not know the cause of chronic kidney failure. Concealing this gap makes it difficult to apply for public funding for the full workup and treatment of diseases that could be diagnosed by biopsy and histological examination, or even genetic evaluation, and delays progress toward the desirable precision medicine. With this letter, we want to call for a joint action to improve the current scenario for a large proportion of CKF patients with unknown primary kidney disease by taking the first step: In the Brazilian Dialysis Survey, we admit our uncertainty about the cause of kidney failure. Recognizing our lack of knowledge is the first step in the pursuit of knowledge.
